# The transition from open to laparoscopic surgery for bilateral inguinal hernia repair: how we did it

**DOI:** 10.1007/s00423-022-02671-w

**Published:** 2022-09-07

**Authors:** Nils Jimmy Hidalgo, Irene Bachero, Carlos Hoyuela, Montserrat Juvany, Jordi Ardid, Antoni Martrat, Salvador Guillaumes

**Affiliations:** 1 Department of Gastrointestinal Surgery, Institute of Digestive and Metabolic Diseases, Hospital Clinic, C. de Villarroel, 170, 08036 Barcelona, Spain; 2 Universitat Internacional de Catalunya, Barcelona, Spain; 3 Department of Surgery, Hospital de Mollet, Mollet, Spain; 4 Department of Surgery, Hospital General Granollers, Granollers, Spain; 5 Department of General and Digestive Surgery, Institute of Digestive and Metabolic Diseases, Hospital Clinic, Barcelona, Spain

**Keywords:** Inguinal hernia repair, Bilateral hernia, Laparoscopy, Minimally invasive surgery, Hernioplasty, Surgical training

## Abstract

**Purpose:**

To describe the transition process from open repair (OR) to laparoscopic repair (LR) of bilateral inguinal hernia in a small basic general hospital

**Methods:**

We describe the technical details and training strategy used to facilitate the transition to systematic LR of bilateral inguinal hernia. We conducted a retrospective analysis of prospectively collected data from all patients undergoing bilateral inguinal hernia repair between January 2017 and December 2020. We analysed the evolution of LR and compared the surgical outcomes: complications, acute pain (24 h), chronic pain (> 3 months), and recurrence (1 year) of the patients operated on by OR and LR.

**Results:**

We performed 132 bilateral inguinal hernia repairs, 55 (41.7%) ORs, and 77 (58.3%) LRs. A significant difference was observed in the choice of LR over time (2017: 9%, 2018: 32%, 2019: 75%, 2020: 91%, *p* < 0.001). The mean operative time was shorter in the OR group than in the LR group (56 min vs. 108 min, *p* < 0.001). However, the operative time of the LR decreased over the years. No significant differences were observed in complications or recurrence. LR was associated with lower acute postoperative pain at 24 h (2.2 vs. 3.1 points, *p* = 0.021) and lower chronic groin pain than OR (1.3% vs. 12.7%, *p* = 0.009).

**Conclusion:**

A structured and systematized training process made the transition from OR to LR of bilateral inguinal hernias feasible and safe in a small basic general hospital. This transition did not increase complications or recurrence. Additionally, LR was associated with a decrease in postoperative pain and chronic groin pain.

## Introduction

Inguinal hernia repair has evolved from herniorrhaphy techniques, which presented a high recurrence rate [1], to tension-free repair using a mesh, as described by Lichtenstein [2], to laparoscopic approaches such as transabdominal preperitoneal laparoscopic repair (TAPP) and totally extraperitoneal repair (TEP) in recent years [, 3, 4]. Studies conducted regarding the laparoscopic approach in inguinal hernia repair describe several benefits, including less postoperative pain and a shorter recovery period [, , 5, 6, 7]. The choice of procedure depends on the surgeon’s skills, patient’s comorbidities, size of the hernia, and available resources [8].

The current international guidelines recommend a laparoscopic approach for bilateral groin hernia repair if surgical expertise and resources are available [, , , , 7, 9, 10, 11, 12]. However, the adoption of the laparoscopic approach has been reported to be very low in Spain (5.7%) [13], and in other countries such as the USA, it reaches 37.8% [14]. The learning curve and cost are the main limitations for choosing laparoscopy for inguinal hernia repair [, , 15, 16, 17]. It is generally accepted that laparoscopic inguinal hernia repair is a demanding procedure requiring advanced laparoscopic surgery skills and a considerable learning curve [18].

In Spain, 74.4% of patients are treated under the coverage of the public health system, and hernia repair is a highly prevalent procedure, with a significant waiting list in some areas. Therefore, the health system often incentivizes the number of procedures and lower cost per session over quality [19]. The additional cost of laparoscopic repair is approximately 500 euros per case [20]. In Spain, only highly specialized centres perform significant percentages of inguinal hernia repairs by laparoscopy, despite the formal indication of this approach, especially in bilateral inguinal hernias [13].

Hospital Plató is currently part of the Hospital Clínic of Barcelona, a large tertiary hospital. However, it has been for many years, and as of January 2021, a small basic general hospital with a high workload in abdominal wall surgery. Similar to most basic general hospitals in Spain, Hospital Plató has not conducted a significant number of laparoscopic inguinal hernia surgeries.

Our study aimed to describe the technical details and training strategy used to facilitate the transition from open repair (OR) to systematic laparoscopic repair (LR) of bilateral inguinal hernia; our experience may be is useful for centres just starting to implement laparoscopic hernia repair. Our goal is mostly descriptive, with no attempt to compare the open and laparoscopic groups beyond demonstrating that laparoscopy did not worsen our outcomes.

## Materials and methods

### A) The structured and systematized process used to do the transition

Until 2017, our standard inguinal hernia repair technique was the open Lichtenstein technique, fixed the mesh with glue, as described by us in previous studies [21]. We continued to use the open Lichtenstein technique in the transition period, progressively replacing it.

**Fig. 1:**
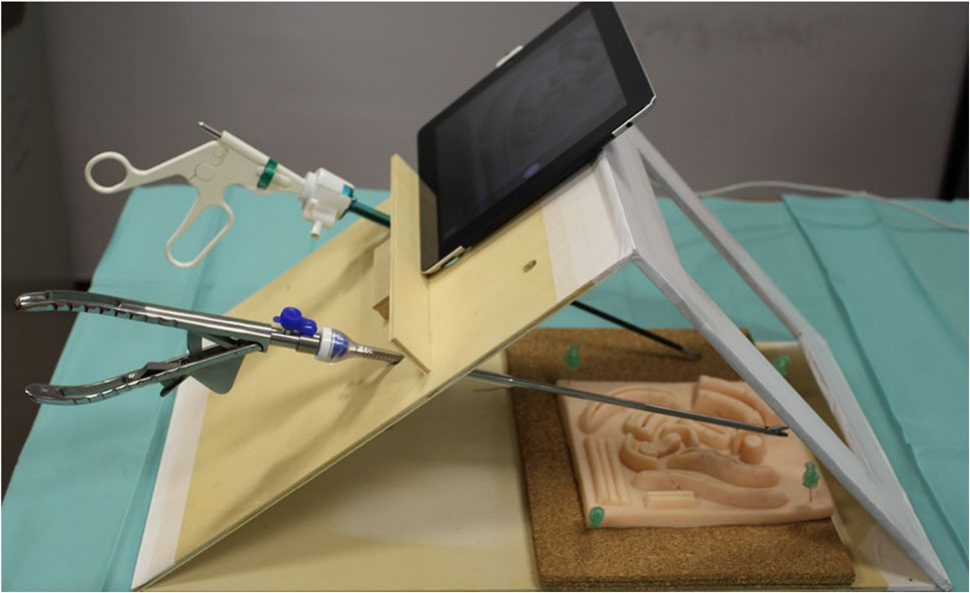
Laparoscopic homemade trainer

The key points used to facilitate the transition to laparoscopic repair were as follows:

1. Selection of the technique. After a bibliographic review, we concluded that TEP and TAPP are equally safe and effective [, , , , 9, 22, 23, 24, 25]. We chose the TAPP technique because of the greater familiarity with this approach in surgeons with long experience in noncomplex laparoscopic surgery. However, we performed two surgeries using the TEP technique during the beginning of the transition to laparoscopic surgery.
2. Selection of the mesh and the mesh fixation system. The cost of certain types of mesh and the fixation system can be limiting factors in the implementation of laparoscopic hernia repair. Simple polypropylene meshes are inexpensive, and fixating them with glue is an inexpensive, safe and widely studied method [, 21, 26]. For this reason, we chose a polypropylene mesh (60 g/m^2^) and fixed it with a tissue adhesive based on n-butyl-cyanoacrylate (Histoacryl. B. Braun surgical SA. Rubí. Barcelona). Standard polypropylene mesh (≥ 50 g/m^2^) decreases the risk of recurrence and is the most cost-effective alternative compared to lightweight mesh (< 50 g/m^2^) [27]. We initially used a vascular catheter to apply the glue as described by Kukleta [26]. From 2019 onward, we started using a laparoscopic applicator provided by the same glue manufacturer.
3. Understanding the preperitoneal anatomy. Another difficulty related to the laparoscopic approach is the greater surgical complexity associated with identifying the “new” anatomy of the posterior inguinal wall, which is not the usual scenario for general surgeons. We reviewed the literature that describes the anatomy of and surgical technique for posterior access by laparoscopy of the groin region. Recent articles confirm that understanding the fascial structures of the groin and neuroanatomy in this region is necessary to obtain good results and avoid complications [, , , , 28, 29, 30, 31, 32]. We also attended training sessions at other hospitals, where we receive theoretical training and observe live surgeries.
4. Training suturing techniques. We built our own simulators for training on knots and suture techniques (Fig. 1). The two surgeons involved initially had little prior experience with laparoscopic sutures, and they spent approximately 100 h training on sutures and knots to fix this problem. In a few weeks, this training helped decrease peritoneum suturing times. Using barbed sutures also helped in diminishing suturing times.
5. Learning by tutor-guided surgery. The surgeries were performed by four surgeons with no prior experience performing laparoscopic inguinal hernia repair. In the first part of the evaluated period, only two of these surgeons participated, and later they tutored the other two surgeons. The two initially involved surgeons attended operations in other hospitals, including a specific TAPP training course. Our first 20 surgeries were supervised by a surgeon with previous experience in TAPP repair.
6. Systematizing the procedure. We established a strict standardization of the operating technique. We considered and applied the technical key points described by Bittner and other authors [, , , 28, 33, 34, 35].

TAPP repair was performed under general anaesthesia. The patient was placed in a supine Trendelenburg position. A urinary catheter collapses the bladder to reduce the risk of bladder injury; it is recommended when technical difficulties or prolonged surgical time are expected [7]. We considered it useful for us in this phase of transition to laparoscopic surgery. However, its routine use is not recommended due to the risk of cystitis, urinary retention or haematuria [36].

Abdominal insufflation was performed with a Veress needle inserted through a supraumbilical incision. We used one 11 mm trocar for the 30° optic at the umbilicus, a 5-mm trocar at the left flank and an 11 mm trocar at the right flank to enter the mesh, gauze swabs and needles for suturing.

We avoided removing adhesions between the bowel or the omentum and the peritoneum near the hernia sac since this would increase the risk of bowel lesions or bleeding.

We opened the peritoneum at the anterosuperior iliac spine and dissected this area by traction of the peritoneum and countertraction of the preperitoneal fat. Fatty tissue in the preperitoneal space should be kept in contact with the abdominal muscle and not with the peritoneum (less risk of nerve damage) [34]. We used blunt dissection at the medial space (Retzius) until identifying the pectineal ligament (Cooper’s) and the pubic symphysis.

For medial hernias, we mobilized the hernia content from the transversalis fascia by traction and countertraction and then inverted and fixed the “pseudosac” to the pubic zone or the rectus muscle with a barbed suture. This inversion and fixation of the fascia transversalis reduces the frequency of occurrence of serohaematoma without an increase in postoperative pain[37].

For lateral hernias, we separated the sac from the cord structures by blunt dissection or with electrocoagulation of the dense adhesions. In complicated cases with a large and severely adhered hernia sac, it is recommended to cut the sac at the level of the inner inguinal ring [34]. We looked for preperitoneal fat herniated through the deep inguinal ring, the so-called lipoma of the cord. When found, we reduced it.

We reduced the peritoneum 4–5 cm below the ileopubic path and used 12 × 15 cm mesh on each side, overlapping 1 cm over the pubic symphysis. We fixed the mesh with glue and closed the peritoneum with a running barbed suture. No drains were used.

### B) Study design

We conducted a retrospective analysis of prospectively collected data from all patients undergoing bilateral inguinal hernia repair between 2017 and 2020. Data collection started in January 2017 when we began a strategy to facilitate the transition to laparoscopic repair in bilateral inguinal hernia.

Inclusion criteria: patients with bilateral inguinal hernia repairs (open and laparoscopic approach) performed between January 1st, 2017 and December 31st, 2020.

Exclusion criteria: patients receiving emergency hernia repair and patients who did not have a postoperative follow-up of at least 1 year.

In our study, the surgeries performed by the laparoscopic approach were performed by four surgeons with experience in noncomplex laparoscopic surgery but without previous experience in laparoscopic inguinal hernia repair. Open repairs were performed consecutively by a single team of two surgeons.

### C) Variables analysed

We collected demographic data for all study patients, including age, sex, and body mass index (BMI). Previous diseases such as high blood pressure, diabetes mellitus, cardiovascular disease (myocardial infarction, heart failure, cardiac arrhythmia), chronic lung disease (obstructive or hypertensive pulmonary disease), liver disease, and obesity were collected. The anaesthetic risk was measured using the American Society of Anesthesiologists (ASA) classification system[38].

We described the characteristics of the inguinal hernia according to the European Hernia Society (EHS) classification [39]. In addition, we collected data on whether the hernia was recurrent and the patient had a history of previous lower abdominal surgery. We also collected the reasons for choosing OR (surgeon with no experience, medical contraindication, hernia characteristic, or patient decision).

The outcomes collected were intraoperative complications, conversion of laparoscopic procedures to open surgery, surgical time, early reintervention, and postoperative complications. The postoperative complications collected were seroma, defined as a localized fluid collection identified by physical or ultrasound examination at the surgical site; haematoma, defined as a collection of blood outside of blood vessels; wound infection, defined as the presence of superficial, deep, or organ space infection; and urinary retention, defined as the need for insertion of a urinary catheter for failure to void. We reported the severity of the postoperative complications according to the Clavien–Dindo classification [40]. Other data collected were postoperative pain at 24 h measured by a 0–10-cm visual analogue scale (VAS), hospital stay measured from admission to hospital discharge, chronic groin pain defined as pain occurring more than 3 months after surgery using a 0–10-cm VAS, and hernia recurrence diagnosed by physical examination or ultrasound in the postoperative follow-up (1 year).

### D) Ethics

Our local ethics committee approved the present study and the retrospective database (HCB/2021/0946).

### E) Statistical analysis

We performed the Kruskal–Wallis test to analyse quantitative and ordinal variables and the linear-by-linear association test for binary qualitative variables to analyse data by year. To compare the open and laparoscopic surgery groups, we performed the chi-square test or Fisher’s exact test to analyse qualitative variables and Student’s t test or the Mann–Whitney *U* test to analyse quantitative variables. A linear regression model was used to analyse the evolution of operative time throughout the series. Statistical significance was established at *p* < 0.05. Statistical analysis was performed using the commercial software SPSS version 20.0 (IBM Corp. in Armonk, NY).

## Results

The study included 132 bilateral inguinal hernia repairs (264 individual hernioplasties) and excluded four patients (one patient for emergency hernia repair and three for incomplete follow-up). The distribution according to sex was: 118 men (89.4%) and 14 women (10.6%), with a mean age of 60.7 ± 12.3 years. During this period, 55 patients (41.7%) were operated on by OR, and 77 patients (58.3%) were operated on by LR. Table 1 shows the baseline characteristics of the total cohort distributed by year. Only 5.3% of bilateral inguinal hernia repairs were performed as outpatient surgery. We also performed 82 laparoscopic unilateral inguinal hernia repairs during the same period. However, it was not the procedure of choice for our hospital due to costs.

**Table 1:** Characteristics, surgery, and results during the period 2017–2020

Year	Total	2017	2018	2019	2020	*p value*
	*N* = 132	*N* = 22	*N* = 25	*N* = 63	*N* = 22	
Age, years						
Mean ± SD	60.7 ± 12.3	57.1 ± 14.8	58.4 ± 10.5	62.1 ± 13.2	63.1 ± 7.7	0.288
Gender, *n* (%)						
Male	118 (89%)	21 (96%)	24 (96%)	53 (84%)	20 (91%)	0.236
Female	14 (11%)	1 (4%)	1 (4%)	10 (16%)	2 (9%)	0.236
BMI						
Mean (SD)	25.8 ± 4.1	24.9 ± 4	26.2 ± 4.2	25.9 ± 3.6	26.3 ± 5.2	0.506
ASA, *n* (%)						0.149
I	39 (30%)	7 (32%)	8 (32%)	7 (27%)	7 (32%)	
II	85 (64%)	14 (64%)	17 (68%)	41 (65%)	13 (59%)	
III	8 (6%)	1 (4%)	0 (0)	5 (8%)	2 (9%)	
Surgery, *n* (%)						
Open	55 (42%)	20 (91%)	17 (68%)	16 (25%)	2 (9%)	< 0.001
Laparoscopic	77 (58%)	2 (9%)	8 (32%)	47 (75%)	20 (91%)	< 0.001
Postoperative complications						
Clavien–Dindo						0.155
I	9 (7%)	3 (14%)	0 (0)	12 (19%)	1 (5%)	
II	3 (2%)	2 (9%)	2 (8%)	2 (3%)	0 (0)	
Length of stay, days						
Mean (SD)	1.1 ± 0.8	1.1 ± 1.4	1 ± 0.5	1.2 ± 0.7	1	0.492
Chronic Groin Pain, n (%)	8 (6%)	3 (14%)	2 (8%)	3 (5%)	0 (0)	0.048

*BMI*, body mass index; *ASA*, American Society of Anesthesiologists classification; *SD*, standard deviation

### Trends in laparoscopic approaches during the study period

**Fig. 2:**
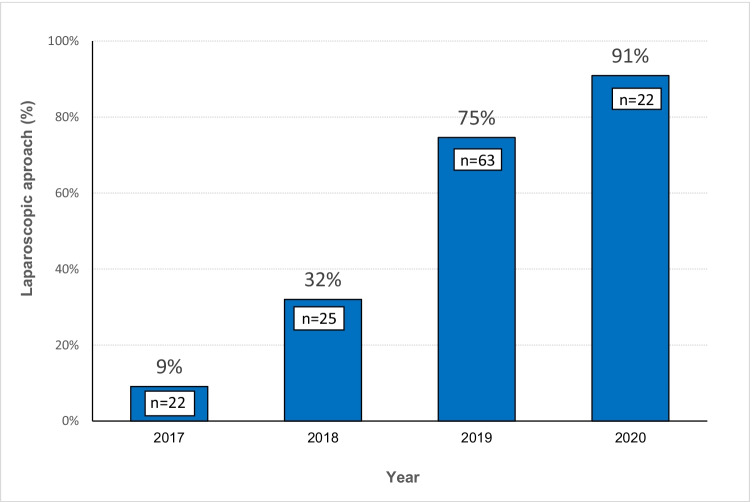
The proportion of bilateral inguinal hernia repair by laparoscopic access in 2017, 2018, 2019, and 2020

We observed a significant difference in the choice of laparoscopic access in the study period (2017: 9%, 2018: 32%, 2019: 75%, 2020: 91%) and found a significant trend (*p* < 0.001) (Fig. 2). As a result of this change, we did not observe differences in postoperative complications classified according to the Clavien–Dindo classification or length of hospital stay.

The reasons for choosing open repair during the study period are described in Table 2. In 2017, the main reason for choosing open access was the surgeon’s lack of experience in laparoscopic inguinal hernia repair (i.e., performing 100% open repairs), which progressively decreased until 2020, when this factor did not exist. In 2019 and 2020, the reason for choosing OR was medical contraindication, hernia size, and the patient’s decision.

**Table 2:** Analysis of the reasons for open surgery during the period 2017–2020

Open surgery reasons	2017 *N* (20)	2018 *N* (17)	2019 *N* (16)	2020 *N* (2)
Surgeon with no experience*, *N* (%)	20 (100)	17 (100)	6 (37.5)	0
Medical contraindication (COPD, or others), *N* (%)	0	0	6 (37.5)	1 (50)
Big (inguinoscrotal) hernia, *N* (%)	0	0	2 (12.5	1 (50)
Recurrent hernia, *N* (%)	0	0	0	0
Patient decision, *N* (%)	0	0	2 (12.5)	0

^*^Surgeon with no experience in laparoscopic inguinal hernia repair

*COPD*, chronic obstructive pulmonary disease

### Demographic and morbidity characteristics

When analysing the cohort divided into two groups according to approach, we found no significant differences in age, sex, BMI, anaesthetic risk (ASA score), or hernia size (Table 3). Moreover, no significant differences were found in previous diseases between the groups, except in the proportion of lung disease, which was higher in the open group than in the laparoscopic group (26% vs. 3%, *p* < 0.001).

**Table 3:** Patient characteristics by open or laparoscopic bilateral inguinal hernia repair

	Open	Laparoscopic	*p value*	OR (95% CI)
	*N* = 55	*N* = 77		
Age, years				
Mean ± SD	59.9 ± 13.5	61.3 ± 11.4	0,520	
Gender, *n* (%)				
Male	52 (95%)	66 (86%)	0.104	0.35 (0.09–1.31)
Female	3 (5%)	11 (14%)	0.104	2.89 (0.77–10.89)
BMI				
Mean ± SD	25.6 ± 4.2	26 ± 4	0.521	
Comorbidity				
Arterial hypertension	22 (40%)	24 (31%)	0.294	0.68 (0.33–1.4)
Diabetes mellitus	6 (11%)	5 (7%)	0.525	0.57 (0.16–1.96)
Cardiac disease	9 (16%)	6 (8%)	0.126	0.43 (0.14–1.29)
Pulmonary disease	14 (26%)	2 (3%)	< 0.001	0.07 (0.01–0.36)
Hepatic disease	1 (2%)	3 (4%)	0.640	2.18 (0.22–21.62)
Obesity	7 (13%)	11 (14%)	0.797	1.14 (0.41–3.16)
Smoking history	35 (64%)	36 (47%)	0.055	0.51 (0.24–1.01)
ASA, n (%)			0.350	
ASA I	15 (27%)	24 (31%)		
ASA II	35 (64%)	50 (65%)		
ASA III	5 (9%)	3 (4%)		
Recurrent_Repair	9 (16%)	6 (8%)	0.126	0.43 (0.14–1.29)
Hernia size (EHS), n (%)			0.174	
Grade I (< 1.5 cm)	3 (6%)	5 (7%)		
Grade II (1.5–3 cm)	36 (65%)	38 (49%)		
Grade III (> 3 cm)	16 (29%)	34 (44%)		
Previous lower abdominal surgery	12 (22%)	14 (18%)	0.605	0.79 (0.34–1.89)

*BMI*, body mass index; *ASA*, American Society of Anesthesiologists classification; *SD*, standard deviation; *EHS*, European Hernia Society; *OR*, odds ratio; *95% CI*, 95% confidence interval

### Surgical outcomes

**Fig. 3:**
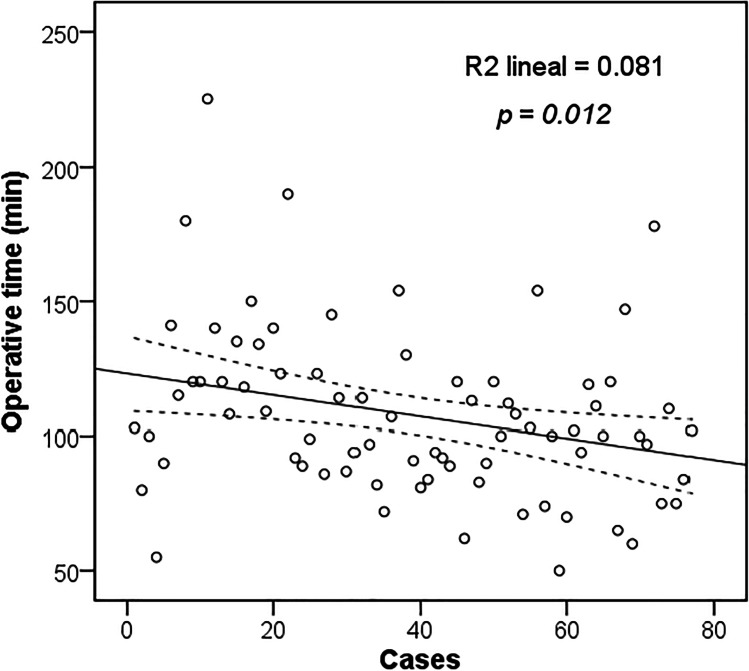
Operative time in consecutive cases of patients with laparoscopic approach. Linear regression analysis shows a significant decrease in operative time (*p* = 0.012)

The surgical outcomes are summarized in Table 4. The mean surgical time was shorter in the open group (56 min vs. 108 min, *p* < 0.001). Figure 3 shows how the operating time significantly decreased over the years in the laparoscopic group (*p* = 0.012). There were no intraoperative complications, conversions to open surgery, or need for reoperation.

**Table 4:** Outcomes by open or laparoscopic bilateral inguinal hernia repair

	Open	Laparoscopic	*p value*	OR (95% CI)
	*N* = 55	*N* = 77		
Surgical technique, n (%)				
Lichtenstein/Lichtenstein	49 (37.1%)			
Lichtenstein/Nyhus	1 (0.8%)			
Nyhus/Nyhus	2 (1.5%)			
Lichtenstein/Plug	1 (0.8%)			
Plug/Plug	1 (0.8%)			
Stoppa	1 (0.8%)			
TAPP		75 (56.8%)		
TEP		2 (1.5%)		
Operative time (min)				
Mean ± SD	56 ± 14	108 ± 31	< 0.001	
Intraoperative complication, *n* (%)	0 (0)	0 (0)		
Conversion to open, *n* (%)	-	0 (0)		
Surgical reintervention, *n* (%)	0 (0)	0 (0)		
Postoperative complications, *n* (%)				
Hematoma	3 (5.5%)	0 (0)	0.07	
Urinary retention	2 (3.6%)	1 (1.3%)	0.570	0.34 (0.03–3.94)
Seroma	1 (1.8%)	12 (15.6%)	0.009	9.96 (1.25–79.13)
Wound infection	3 (5.5%)	0 (0)	0.07	
Clavien–Dindo, *n* (%)			0.743	
I	3 (5.5%)	13 (16.9%)		
II	5 (9.1%)	1 (1.3%)		
VAS 24 h, mean ± SD	3.1 ± 2.2	2.2 ± 1.8	0.021	
Length of stay, days				
Mean ± SD	1.1 ± 1.1	1.1 ± 0.4	0.129	
Re admission 30 days	0	0		
Chronic groin pain (≥ 3 m), *n* (%)	7 (12.7%)	1 (1.3%)	0.009	0.09 (0.01–0.75)
Hernia recurrence, *n* (%)	2 (3.7%)	2 (2.6%)	1.000	0.69 (0.09–5.08)

*TAPP*, transabdominal preperitoneal; *TEP*, totally extraperitoneal; *SD*, standard deviation; *VAS*, visual analogue scale; *OR*, odds ratio; *95% CI*, 95% confidence interval

Postoperative complications were minor (Clavien–Dindo grade I or II), with no significant differences between the groups. However, patients in the laparoscopic group experienced a higher seroma rate (15.6% vs. 1.8%, *p* = 0.009).

Analysis of the VAS score demonstrated a small reduction in acute postoperative pain 24 h after surgery in laparoscopic repair patients (3.1 points vs. 2.2 points, *p* = 0.021). Chronic groin pain (≥ 3 months after surgery) was significantly lower in the laparoscopic group (1.3% vs. 12.7%, *p* = 0.009).

There were no differences in hernia recurrence between the two groups during follow-up (1 year).

## Discussion

We observed that the transition towards a laparoscopic approach for bilateral inguinal hernia repair, when following a systematized strategy, is safe and feasible in a small basic general hospital. This transition did not increase postoperative complications but did imply a decrease in immediate postoperative pain and chronic groin pain.

In the period 2016–1018, the global rate of LR for inguinal hernia in Spain was 5.7%, reaching 17.9% for bilateral hernia; only in one of the 51 provinces of the country was the rate of LR for bilateral hernia over 50% [13]. In our study, the choice of LR for bilateral hernia increased from 9.1% in 2017 to 90.9% in 2020.

Currently, international guidelines approve both open and laparoscopic approaches when performing unilateral inguinal hernia repair. At the same time, they recommend using the laparoscopic approach in bilateral inguinal hernia repair [, , , 7, 10, 11, 12]. However, LR has been slow to gain acceptance, and this could be due to relative contraindications such as suitability for general anaesthesia, a history of previous abdominal surgery, the size of the hernia, lack of standardization of the surgical technique, the learning curve, and some infrequent but serious reported complications [41]. Additionally, from an individual hospital point of view, the laparoscopic approach is more expensive, even though it is more cost-effective (especially for bilateral hernias) from the socioeconomic perspective due to the quicker return to work [, 11, 20].

We had no intraoperative complications in either group, and none of the laparoscopic procedures were converted to open surgery. In a large series of 2880 TAPP repairs of bilateral hernias, intraoperative complications were described in only 0.24% of the procedures [42].

A recent meta-analysis comparing TAPP and Lichtenstein repairs for unilateral hernias found no significant differences in postoperative complications [43]. Other studies looking at complications in bilateral hernias have shown a lower complication rate with the laparoscopic approach [, 44, 45]. Our study did not find significant differences in the rate of complications classified according to Clavien–Dindo. However, we found a higher frequency of seromas in the laparoscopic group, probably related to the nonfixation of the pseudosac in our first cases. The incidence of postoperative seroma was reduced by systematic fixation of the pseudosac with a barbed suture. The transition to the laparoscopic approach in our hospital did not increase total complications in the analysed years.

Some prospective randomized trials [, , 45, 46, 47] have associated the laparoscopic approach with less postoperative pain, less need for analgesics, and earlier return to work compared to the corresponding outcomes of the open technique. We observed a significantly lower VAS score at 24 h in the laparoscopic group than in the open approach group. We also found a lower frequency in the laparoscopic group when evaluating chronic groin pain. Since tension-free mesh techniques have lowered the recurrence rate, lately, attention has focused on reducing chronic groin pain, which is a problem that affects up to 30% of patients undergoing open repair of inguinal hernia [, 48, 49]. In this regard, the evidence describes less chronic pain after laparoscopic repair compared to open hernia repair [, , 43, 50, 51].

As stated before, with tension-free mesh repair, the recurrence rate of inguinal hernia decreased to 1–4%, and Lichtenstein repair became the gold standard of inguinal hernia repair [52]. The results of long-term randomized controlled trials comparing laparoscopic and open repair of unilateral inguinal hernia show similar recurrence rates (open; 3–5% vs. laparoscopic; 2–4%) [, 53, 54]. However, its incidence in bilateral hernias is not well understood because few studies with heterogeneous populations have been published. Feliu et al. [44] reported a recurrence rate of 1.3% for TEP and 3.8% for the Lichtenstein technique after a bilateral procedure. Furthermore, establishing the recurrence rate is difficult without a national registry to track patients with inguinal hernia. We found no differences in the recurrence rate between open and laparoscopic approaches (3.7% vs. 2.6%).

The operative time for the laparoscopic approach compared to the open approach is longer for unilateral hernias [43]. However, when comparing operative times in bilateral hernias, this difference is smaller, and some studies even report shorter operative times in laparoscopic repairs [44]. In our study, the operative time of the laparoscopic group was longer than that of the open group, mainly due to the learning curve, but it showed a significant decrease in the following years. We believe the operative time will continue to decrease in the coming years if we improve our laparoscopic skills.

Our experience shows a way to transition to laparoscopic repair in inguinal hernias in basic general hospitals. The difficulty of the learning curve is not as great in surgeons with previous experience in laparoscopic surgery. Additionally, using simple polypropylene mesh, fixing the mesh with glue, and using nondisposable instruments can reduce costs.

The present study has limitations commonly related to a retrospective study design and contained certain biases, including age and comorbidities. In the initial phase of the study, the selection bias implicit in the learning curve led to the avoidance of laparoscopic surgery in patients with large hernias. Another limitation is the length of our follow-up period since postoperative data could change after 1 year. Additionally, due to the retrospective nature of the study, some complications that could appear before the 1-year follow-up visit might not have been correctly collected. Nonetheless, the results are useful for analysing the implantation process of this new surgical technique in our hospital.

## Conclusions

The transition from open bilateral inguinal hernia repair to the laparoscopic approach is feasible and safe. This process does not increase intraoperative or postoperative complications or recurrence rates. Additionally, the laparoscopic approach is associated with a decrease in postoperative pain and chronic groin pain. We hope that the description of our experience can be useful to surgeons who want to begin their transition to laparoscopic surgery in inguinal hernia repair. The key points are knowledge of the anatomy, the details of the technique and its systematization, and, if necessary, training in suturing techniques.
